# Unraveling Nanoplastics–Enzyme Interactions: Physicochemical, Structural, Functional, and Cell Biological Characterization of α‑Amylase–Nanoplastics Complexes

**DOI:** 10.1021/acs.langmuir.6c00976

**Published:** 2026-07-07

**Authors:** Holger Sieg, Franziska Ott, Linda Böhmert, Stephan Drusch, Andreas F. Thünemann, Sascha Rohn, Helena Kieserling

**Affiliations:** † Department of Food and Feed Safety in the Food Chain, Unit Novel Foods, GMOs, Food Additives, Flavourings and Feed Additives, German Federal Institute for Risk Assessment(BfR), Max-Dohrn-Str. 8-10, Berlin 10589, Germany; ‡ Department of Food Chemistry and Analysis, Institute of Food Technology and Food Chemistry, 26524 Technische Universität Berlin, Kaiserin-Augusta-Allee 14, Berlin 10553, Germany; § Department of Food Technology and Material Science, Institute of Food Technology and Food Chemistry, Technische Universität Berlin, Straße des 17. Juni 135, Berlin 10623, Germany; ∥ Bundesanstalt für Materialforschung und -prüfung (BAM), Unter den Eichen 87, Berlin 12205, Germany

## Abstract

The topic of micro- and nanoplastics received significant attention in recent decades due to increasing environmental exposure, strong public perception, and emerging health concerns. While knowledge regarding detection and material characteristics has improved, the understanding of impact on cells remained unclear. As biological effects are initially caused by molecular interactions, consequently direct interactions with biomolecules, such as enzymes, are of particular relevance. In this occasion, effects may vary depending on the plastic type and particle properties. The specific aim of this study was to characterize the direct molecular interactions by means of selected model proteins and a variety of different nanoplastic particles. Therefore, the aim of the study was to exemplarily characterize α-amylase’s (as a model enzyme) interactions with different nanoplastics and the resulting effects on enzyme structure and function, as well as cellular responses. The properties of the α-amylase–nanoplastic mixtures were analyzed using dynamic light scattering (DLS), Fourier-transform infrared spectroscopy (FTIR), fluorescence spectroscopy, and Phadebas amylase activity test. Additionally, Caco-2 cells were used as a model system for the human intestinal barrier and exposed to these complexes to evaluate cellular uptake through flow cytometry, microscopy, and viability testing. All applied nanoplastics interacted with α-amylase, forming complexes with adsorption affinities that depended on the particle type (PP ≫ PE > PET ≫ PLA). FTIR and fluorescence analyses showed particle-specific structural changes. Despite these differences in structural response, concentration-dependent enzyme inhibition was measurable, depending on the particle type. Uptake studies on Caco-2 cells indicated no internalization or cytotoxicity. These findings suggest that nanoplastics influence the enzyme structure and function based on their chemical properties, offering new insights into direct enzyme–nanoplastics interactions and their potential impacts on enzymes and cells.

## Graphical abstract

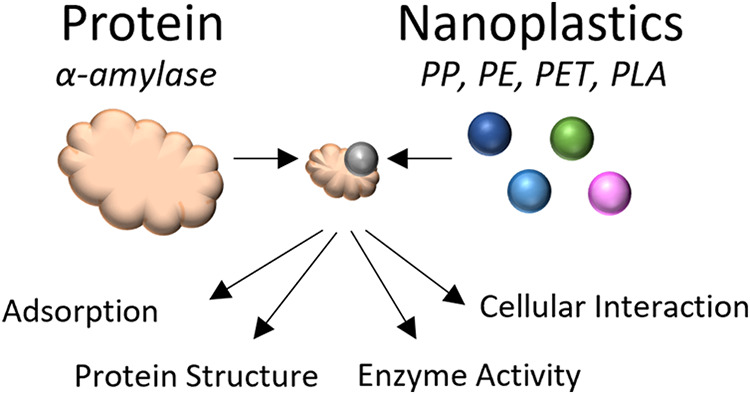

## 1 Introduction

Micro- and nanoplastics (MNP) have been intensively studied in the last 20 years,^1^ driven by high public and scientific awareness of possible impacts on the environment and human health.^2^ With the progress of detection technology, methods have improved, and consequently, MNP were found in food and many products of daily life.^1,3^ Whereas the knowledge about abundance and exposure increased, the mechanistic understanding of effects remains limited, causing data gaps regarding toxicological modes of action.^4,5^ This lack of mechanistic insight is likewise a major reason why a comprehensive risk assessment of MNP for human health protection is still not possible.

Plastics released into the environment persist, degrade, or fragment into smaller particles due to various environmental factors.^6^ Although no universal definition exists, we defined for enhancing clarity of the present study, microplastics as particles from 1 μm to 5 mm, and nanoplastics as particles smaller than 1 μm, accordingly.^7^ Nanoplastics should not be confused with nanoparticles, which are defined as particles between 1 and 100 nm in size,^8^ and nanoplastics exhibit fundamentally different properties, rather than being just smaller microplastics or classical nanoparticles.^9^ Plastic materials are described as generally persistent and chemically inert,^10^ but this does not preclude interactions with biomolecular structures. These interactions, however, are not yet finally understood. Specifically for each material and physichochemical conditions, these interactions can vary from pure physical adsorption, the formation of a so-called protein corona up to more profound alterations in protein structure and function, recently reviewed by Zhang and colleagues.^11^ This protein interaction can have a significant impact on interactions with cells and biological functions.^12^

As described in various adverse-outcome pathways (AOPs), every biological impact is initiated by a distinct molecular initiating event.^13^ A cascade of key events then propagates this initial trigger, eventually leading to an adverse health outcome. For MNP, these molecular initiating events remain largely unclear. To date, no causal link has been established between MNP exposure and clearly evident health impact. Scientific hypotheses suggest possible direct interactions between MNP and cellular components, such as membranes, filaments, and above all, with proteins. Proteins, being the initial molecular targets in the cell, can interact with MNP in various ways.^14,15^

Thereby, protein adsorption is crucial for understanding potential toxic effects of MNP.^16^ Recently, we confirmed interactions of MNP with the model protein β-lactoglobulin and showed protein adsorption, causing structural changes in β-lactoglobulin.^17^ Given that many proteins show enzymatic functions—particularly when MNP enter the gastrointestinal tract—it needs to be questioned whether the binding of MNP to proteins might alter enzymatic activity. Such particle–enzyme complexes could, in turn, impact cellular uptake and overall cell function upon contact with cells.

The particular aim of the present study is to gather combined information on physicochemical particle properties, their impact on enzyme structure and activity, and furthermore their effects on biological (*in vitro*) cell systems. Accordingly, this study was structured in three parts: first, the physicochemical assessment of MNP–enzyme adsorption; second, the effects of the adsorption on enzyme structure and activity; and third, the impact of MNP and their enzyme complexes on a selected *in vitro* cell system of human intestine. It was focused on nanoplastics because their larger surface area compared to microplastics increases the potential for interactions with enzymes. Moreover, it was assumed that due to their small size, nanoplastics can especially interact with small cavities and catalytic centers, which is likely to hinder enzymatic function. α-Amylase was chosen as a model enzyme due to its ubiquity and essential role in digestion.^18^ Nanoplastics composed of polypropylene (PP), polyethylene (PE), poly­(ethylene terephthalate) (PET), and poly­(lactic acid) (PLA) were selected, as they represent common types of nanoplastics found in the environment and are potentially present in food. Consequently, these nanoplastics are likely ingested and come into direct contact with digestive enzymes in the gastrointestinal tract. Taken together, the present study aimed at investigating how initial molecular interactions between nanoplastics and enzymes can influence the enzyme structure, function, and ultimately cellular responses—thereby providing mechanistic insight into the biological impact of nanoplastics.

## 2 Materials and Methods

### 2.1 Material Synthesis and Preparation

#### 2.1.1 Preparation of α-Amylase Solutions

In the present study, a variety of spectroscopic and analytical assays were performed, each of which, however, required specific concentrations and other prerequisites. The detailed preparation protocols of the required solutions can be found in the Supporting Information (Materials and Methods Section).

#### 2.1.2 Preparation of Nanoplastics

All chemicals were used as received without further purification. Polypropylene (isotactic, Mw ≈ 12,000 g mol^–1^, Mn ≈ 5000 g mol^–1^) was purchased from Sigma-Aldrich (Merck KGaA, Darmstadt, Germany). Polyethylene (Mw ≈ 4000 g mol^–1^, Mn ≈ 1700 g mol^–1^) was purchased from Sigma-Aldrich (Merck KGaA, Darmstadt, Germany). Poly­(ethylene terephthalate) PET Lighter C93 (standard bottle grade) was purchased from Equipolymers S.R.L. (Amsterdam, The Netherlands). Note that the reference material BAM-P206—a PET microplastic powder—was produced from the same product. Acetone (p.a., min. 99.5%, ChemSolute) was purchased from Th. Geyer GmbH & Co. KG (Renningen, Germany). The water for preparation was purified using a Sartorius arium 611 DI purifier. Folded filters of grade 2105 (fast filtering, particle retention 12–15 μm, by LabSolute) were purchased from Th. Geyer GmbH & Co. KG (Renningen, Germany).

The preparation and characterization of PP nanoparticles have been reported in detail.^19^ The same method was applied here for PP, PE, and PET. Briefly, 6.0 g of polymer granules were added to a tall glass beaker, followed by the addition of 115 mL of acetone. The beaker was cooled in an ice bath to prevent acetone evaporation during the preparation. After dispersion, the solution was filtered through a folded filter to remove larger polymer aggregates. Acetone was evaporated until ∼10% of the liquid remained. Then, 115 mL of water was added to the mixture, and the remaining acetone was evaporated, yielding an aqueous dispersion of polymer nanoparticles. The dispersion was then filtered again with a folded filter to remove any particles that might have aggregated during the transfer to water. The remaining dispersions were slightly turbid.

PLA nanoplastics were prepared by nanoprecipitation.^20^ A total of 5.0 g of PLA granules and 5.0 g of Pluronic F127 were placed in a 1 L three-necked round-bottom flask equipped with a stirrer and distillation attachment, following a Claisen adapter connected to a Liebig condenser and a 500 mL one-necked flask. Then, 250 mL of tetrahydrofuran was added to the flask, and the mixture was heated in a water bath at 50 °C while stirring at 650 rpm. Next, 250 mL of ultrapure water was quickly added, forming a milky dispersion with larger aggregates. The dispersion was heated to 55 °C for 1 h, then to 65 °C for 1 h (in a water bath) until no large aggregates remained visible. The apparatus was then insulated with aluminum foil, the water bath was heated to 70 °C, and tetrahydrofuran was distilled off as much as possible. At the end of distillation, the crude product in the distillation flask was filtered into a 250 mL screw-cap glass container using a pleated filter, covered with a dust-free cloth, and left overnight to evaporate any remaining tetrahydrofuran. In dynamic light scattering, the PLA particles exhibit a monomodal particle size distribution with a mean hydrodynamic diameter of 340 nm ± 31 nm and a polydispersity index of 0.2–0.3. Pluronic F127 provides colloidal stability to the PLA particles, enabling the preparation of stable long-term PLA dispersions at 1 wt %. In contrast, no stabilizer was used for the preparation of PP, PE, and PET particles. Hence, stable long-term dispersions were available only at low particle concentrations of 0.004 wt %. It should be noted that varying concentrations of PLA on one side and PP, PE, and PET particles on the other may influence the results presented here, at least in principle. However, we could not prepare PLA and PET­(-Alexa) particles using the same procedure. An overview of the (mean intensity-weighted) hydrodynamic diameters and concentrations of the nanoplastics particles is provided in Table 1.

**Table 1:** Hydrodynamic Diameters *D*_h_ and Selected Concentrations *c* of Nanoplastics (Polypropylene (PP), Polyethylene (PE), Polyethylene Terephthalate (PET), and Polylactic Acid (PLA))

particle type	*D*_h_ [nm]	*c* [mg/L or g/L]
PP	172 ± 8	41 ± 4 mg/L
PE	215 ± 35	27 ± 5 mg/L
PET	128 ± 9	44 ± 2 mg/L
PET-Alexa	200 ± 20	5 g/L
PLA	340 ± 40	10 g/L

### 2.2 Structure and Activity Analysis of α-Amylase

#### 2.2.1 Dynamic Light Scattering (DLS)

The hydrodynamic diameter (*D*_h_) was determined using the Litesizer DLS 500 from Anton Paar GmbH (Graz, Austria). Omega cuvettes were used, and measurements were taken at a backscatter mode at an angle of 175°. The measurements were performed at 25 °C. α-Amylase (solution 1) and the nanoparticle solutions containing PP, PE, PET, or PLA were used. All solutions were first filtered using a 450 nm PVDF filter (Merck Millipore Ltd. Tullagreen, Carrigtwohill, Co. Cork, Ireland). Each sample was measured once in its pure form and once after mixing with α-amylase. The particle solutions and α-amylase (solution 1) were not diluted before measurement. For the mixtures, the particle solutions and α-amylase solution 1 were combined in ratios of 1:1 and 1:10. Each sample was vortexed for 30 s, then 1 mL was added to the Omega cuvette. All measurements were performed in triplicate.

#### 2.2.2 Fourier-Transform Infrared Spectroscopy

FTIR measurements of α-amylase, particles, and mixtures were performed using a Bruker Tensor II spectrometer (Bruker Optics GmbH & Co. KG, Ettlingen, Germany) equipped with a liquid nitrogen-cooled mercury-cadmium-telluride detector and a Bruker BioATR II cell. Measurements were conducted using ultrapure water as background over the range of 1000–3100 cm^–1^ with a resolution of 2 cm^–1^, following 5 min incubation of the samples at 20 °C. Raw spectral data were evaluated and processed with Bruker OPUS Software 7.5, vector-normalized in the Amide I region, and converted to second derivative spectra using the 25-point Savitzky–Golay method. FTIR measurements were performed for each sample in independent triplicate.

#### 2.2.3 Fluorescence Spectroscopy

Measurements of the intrinsic fluorescence of α-amylase, particles, particle–amylase mixtures, and Fe–amylase mixtures were carried out using a Cary Eclipse spectrometer from Agilent Technologies Deutschland GmbH (Waldbronn, Germany) in 3 mL quartz cuvettes with 10 mm optical path length. Samples were excited at 295 nm, and emission spectra were recorded from 300 to 600 nm. Intrinsic fluorescence experiments were performed for each sample in independent triplicate.

#### 2.2.4 α-Amylase Activity Assay

α-amylase activity was analyzed in α-amylase solutions, particle–amylase mixtures, and Fe–amylase mixtures using the Phadebas Amylase Test (Phadebas AB, Kristianstad, Sweden). Measurements were performed using a StellarNet miniature UV/vis spectrometer (StellarNet, Inc., Tampa, FL, USA), and α-amylase activities were calculated from a supplied standard curve. Samples were incubated for 5 min at 37 °C, substrate tablets were added, incubated for 15 min, and reactions were stopped with 0.5 M NaOH (Fisher Scientific UK, Loughborough, England). After centrifugation (5 min, 12,000*g*), 800 μL of a supernatant was measured at 620 nm. Each condition was measured in independent triplicate.

### 2.3 Cellular Effects of Particle–Amylase Mixtures

#### 2.3.1 Cell Cultivation and Sample Preparation

Caco-2 cells were purchased from the European Collection of Cell Culture (ECACC-Nr. 86010202) and used beginning from passage 15 for a maximum of 10 passages after thawing. The cells were kept in DMEM high glucose with 10% fetal calf serum (FCS) and 1% penicillin/streptomycin (P/S) (GE Healthcare GmbH, Düsseldorf, Germany) and cultivated subconfluently. 5000 Caco-2 cells were seeded into each cavity of 96-well plates and xCELLigence E-plates, and 55,000 cells in 12-well format. Proliferating Caco-2 cells were used for experiments 24 h after seeding. For differentiation, in every plate format, the medium was changed every 2–3 days for a differentiation time of 21 days. The particles and their enzyme complexes (using predissolved α-amylase stock solutions) were diluted into a cell culture medium by a factor of 10–1000. As incubation volumes, 100 μL (96-well, proliferating Caco-2), 180 μL (E-plate, proliferating, and differentiated Caco-2), 300 μL (96-well, differentiated Caco-2), and 1 mL (12-well, proliferating Caco-2) were used.

#### 2.3.2 Cell Growth and Cell Viability via Cell Index Measurements

To assess cell growth and viability, cellular impedance was measured using an xCELLigence system (Roche Diagnostics Deutschland GmbH, Mannheim, Germany). Caco-2 cells were seeded in special gold-coated 96-well E-plates and incubated with the test substances over a period of 72 h. For cell growth analysis, proliferating Caco-2 cells (24 h after seeding) were incubated with particle–enzyme complexes. For incubation, the medium was removed and replaced with the testing substances. Cell indices were normalized at a time point 30 min before incubation. For cell viability testing, Caco-2 cells were differentiated for 21 days prior to incubation with particle–enzyme complexes. As positive control, ZnCl_2_ was used in a concentration of 100 μg Zn/mL. Cell indices were determined in triplicate for each experiment, and results are reported as mean ± standard deviation.

#### 2.3.3 Fluorescence Labeling of PET with Alexa-633 Dye

The Alexa-633 dye-labeled PET nanoplastics were prepared by nanoprecipitation.^20^ In a 100 mL beaker, 20 mL of DMSO was heated to 180 °C, and 200 mg of PET powder was added with stirring at 250 min^–1^ using a magnetic stir bar until fully dissolved. Subsequently, 200 mg of Pluronic F127 was added to the PET solution, which dissolved immediately. This was followed by the addition of 0.1 mL of Alexa Fluor 633 in DMSO. The heat source was removed, and the solution was allowed to cool to 110 °C. While stirring, 20 mL of a 2 wt % aqueous Pluronic-127 solution was added in a single step as the temperature decreased to 70 °C. After 15 min, when the temperature reached 50 °C, the product was filtered through a 5–13 μm paper filter. The obtained dispersions were dialyzed with a dialysis membrane (Spectra/Por7 Dialysis Membrane; Pretreated RC Tubing; MWCO of 10^3^ g mol^–1^, Repligen Corporation, Waltham, MA, USA) against water for 10 days, until the dialysate conductivity stabilized.

For determination of the correct excitation and emission wavelengths, absorption and fluorescence measurements were performed using a plate reader (Tecan Group Ltd., Männedorf, Switzerland). Labeled and unlabeled particles and controls were diluted 1:10 into 100 μL of PBS and measured in transparent 96-well plates. Absorption scans were conducted between 230 and 1000 nm wavelengths. To determine the optimal fluorescence wavelengths, excitation and emission scans were performed and showed an optimal response at 610/650 nm (Ex./Em.).

#### 2.3.4 Fluorometric Detection of Particle Uptake

To determine particle uptake, optical and confocal microscopy, as well as fluorescence quantification via plate reader and flow cytometry were applied. For optical microscopy, Caco-2 cells were seeded into 12-well plates (proliferating Caco-2) or 96-well plates (differentiated Caco-2) as described. 24 h after incubation with the testing substances, the medium was removed, cells were washed with PBS, and stored in another 1 mL of PBS (proliferating Caco-2) or 100 μL of PBS (differentiated Caco-2). Measurements were conducted using a high-content screening microscope Cell Discoverer 7 by Zeiss (Carl Zeiss AG, Jena, Germany) with a lens magnification of 5×. Imaging was measured using a brightfield channel (cell morphology) and red fluorescence (631/647 nm Ex./Em.) for particle detection.

For confocal microscopy, Caco-2 cells were seeded into 12-well format on glass plates and differentiated as described before. After differentiation, the cells were incubated with particle–enzyme complexes for 24 h. After washing the plates with PBS, cell nuclei were stained with DAPI (displayed in blue) for 20 min in dark. To further visualize the cytoskeleton, after washing with PBS, the cells were stained with 2 drops/mL PBS ActinGreen 488 ReadyProbes Reagent (Life Technologies Corporation, Carlsbad, USA) for 3 h in the dark at room temperature. The particles were displayed in red color. The excess stain was removed by washing the samples with PBS and distilled water. Kaiser’s glycerin gelatin was used for mounting the samples on microscope slides with cover glasses. After drying the slides overnight at 4 °C, the epithelium was investigated by using the laser scanning confocal microscope LSM 700 (Carl Zeiss AG, Oberkochen, Germany) with three channels: 405/435 nm (DAPI), 488/518 nm (Actin Green), and 639/669 nm (Alexa-633). Images were recorded by measuring Z-stacks spanning through the entire cell layer. Fluorescence quantification was performed using a plate reader (Tecan Group Ltd., Männedorf, Switzerland) in scanning mode. Caco-2 cells were seeded in 12-well (proliferating Caco-2) or 96-well (differentiated Caco-2) plates and cultivated as described before. The medium was removed for incubation with particle–enzyme complexes. After an incubation time of 24 h, the medium was removed and cells were washed twice with PBS. Plate reader measurements were conducted using 610/650 nm wavelength (Ex./Em.) with multiple reads (4 × 4 over each well). Fluorescence intensity values were averaged from at least three independent replicates with calculated mean ± standard deviation. For fluorometric detection via flow cytometry, Caco-2 cells were seeded into 12-well plates (proliferating Caco-2) or 96-well plates (differentiated Caco-2) as described. 24 h after incubation with the testing substances, the medium was removed, cells were washed with PBS, and trypsinized for 5–7 min (proliferating Caco-2) or 40 min (differentiated Caco-2) until they were detached and singularized. Trypsination was stopped using 1 mL of warm serum-containing medium. Cells were centrifuged (300*g*, 5 min) and washed twice with PBS prior to measurement. For measurement, the cells were introduced into a microchannel cuvette to ensure single-cell suspension. A laser beam (640 nm) was directed at the cells, measuring fluorescence intensity (FL4, 675/25 nm). Measurements were run until either 10,000 cells were detected up to a maximum of 2 min for each condition. The mean values of the fluorescence intensity of the cell population were used as a measure for particle uptake. The experiments were performed in at least three independent replicates for each experimental condition. Furthermore, a cell granularity analysis was performed as described before using flow cytometry.^17^

### 2.4 Statistical Evaluation

Data sets were tested for normality using the Shapiro–Wilk test and for homogeneity of variance using the Brown–Forsythe test in GraphPad Prism 9. One-way ANOVA with Tukey’s post hoc test was applied to each data set, with significance defined as *p* < 0.05. Significant differences are indicated in the figures by different letters.

## 3 Results and Discussion

### 3.1 Adsorption of α-Amylase onto Nanoplastics

To investigate the adsorption affinity between α-amylase and nanoplastics, hydrodynamic diameters were analyzed for pure α-amylase, individual nanoplastics (PP, PE, PET, PLA), and mixtures of α-amylase with these nanoplastics. The maximum values of the intensity-weighted size distribution are listed in Table S1. Pure α-amylase shows a broad size distribution weighted by intensity, with the most common sizes between 150 and 700 nm, peaking around 400 nm. This clearly hints at self-aggregation of α-amylase molecules. Additionally, a small peak at about 6 nm was observed, which corresponds to monomeric α-amylase. However, α-amylase mainly does not occur as a well-defined monomer but forms self-aggregations and furthermore enlarged surface modalities due to rather irregular geometry, which does not allow calculations to relate the protein surface with nanoplastics concentrations. This self-aggregation of α-amylase needs to be considered when comparing it to the α-amylase nanoparticle mixtures. A strong increase in the hydrodynamic diameter of the nanoplastics after adding α-amylase indicates a high adsorption affinity of the enzyme for the particle surface. Moreover, an increased hydrodynamic diameter in the mixtures is interpreted as the formation of “nanoparticle–α-amylase complexes”.

The hydrodynamic diameter of pure PP particles and the PP-α-amylase mixture is shown in Figure 1A. The pure PP particles have a maximum at about 200 nm. After adding α-amylase, the size distribution shifts significantly toward large diameters, with a maximum at around 600 nm. Additionally, no traces near the original 200 nm range are detectable, indicating almost complete adsorption of α-amylase to PP particles and, consequently, a high adsorption affinity. This observation is in line with Yang et al., who illustrated in a review that in many systems, nanoparticle surfaces become fully covered by proteins, resulting in the formation of protein–nanoplastics complexes.^21^ In some cases, these further protein–protein interactions can even lead to large agglomerates in which the original individual particles are no longer detectable, as it appeared for the α-amylase–PP mixture in the present study. Given the nonpolar nature of PP, the initial interaction between the enzyme and the particle surface is likely dominated by hydrophobic associations and van der waals forces. Hydrophobic interactions and van der Waals forces were previously described to be drivers of nanoplastics interactions with biomolecules.^22^ The observed behavior is usually accompanied by different noncovalent forces, further including dipole-related interactions, and potentially electrostatic contributions, depending on the surface chemistry of the particles and the local chemical conditions, which is not further specified here.

**Figure 1:**
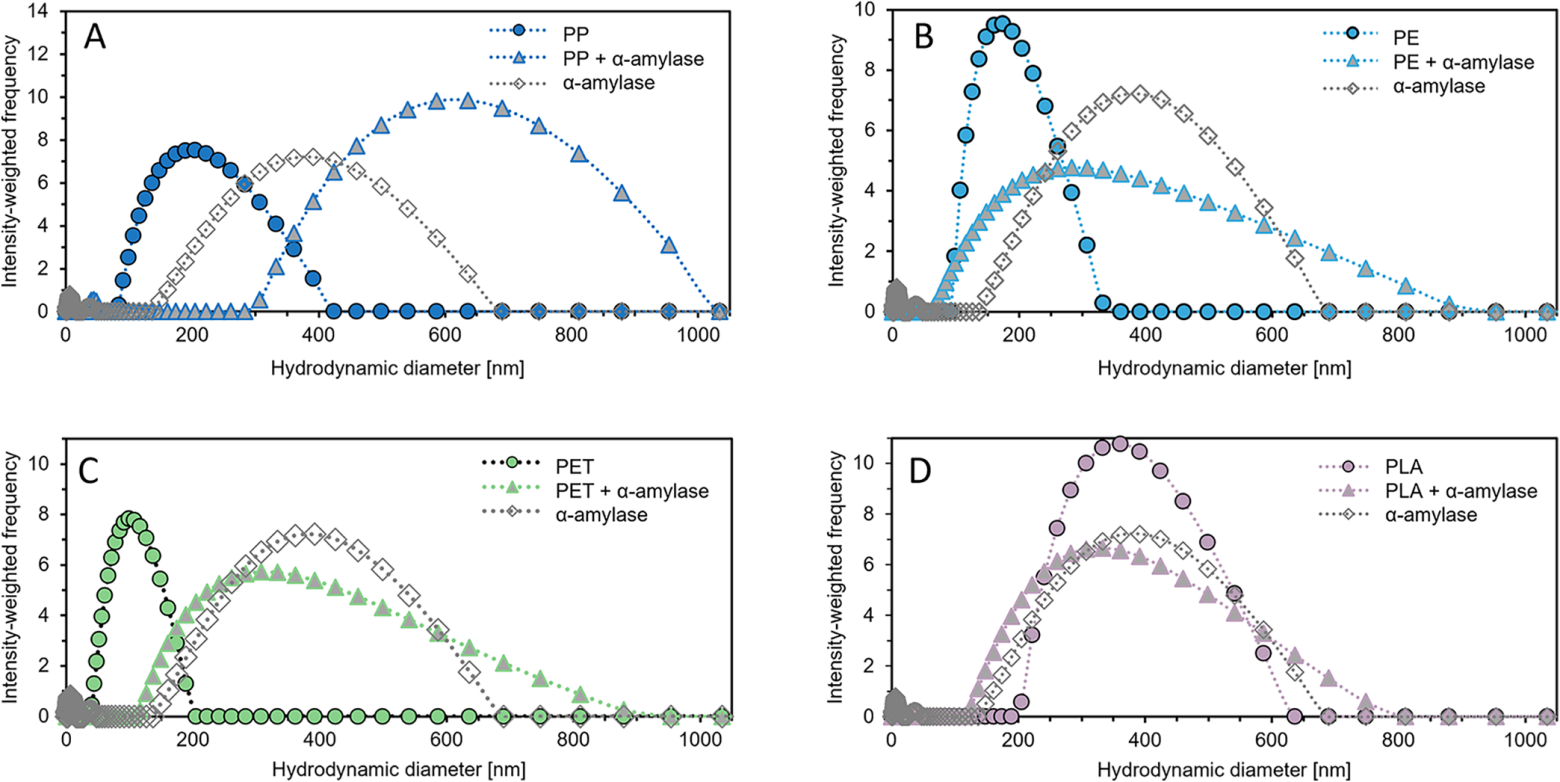
Size distribution of the hydrodynamic diameter of α-amylase, nanoplastics particles, and mixtures thereof. The particles in the respective panels are (A) PP, (B) PE, (C) PET, and (D) PLA.

In Figure 1B, the size distribution of pure PE particles and the PE-α-amylase mixture is shown. The PE particles had a signal maximum at around 180 nm in their pure form. After adding α-amylase, the particle size distribution shifted toward larger diameters, reaching about 850 nm. This trend is in agreement with Wolfram et al., who suggested a high affinity of proteins containing hydrophobic amino acids for nonpolar particle surfaces.^23^ However, compared with PP, some PE particles around 200 nm remained visible, indicating only partial adsorption of α-amylase onto the PE particle surface. This adsorption is also likely governed by hydrophobic associations and van der Waals forces, but with a lower number of binding sites or weaker binding strength compared to PP.

The size distributions of pure PET particles and the PET–α-amylase mixture are shown in Figure 1C. The pure PET particles exhibited a maximum near 100 nm. In the mixture with α-amylase, the size distribution shifted significantly toward larger hydrodynamic diameters, ranging from about 100 to 900 nm. This indicates complete adsorption of α-amylase molecules, as no particles below 100 nm were detected in the region where the pure PET particles were previously present. The maximum of the PET–α-amylase mixture occurred at about 300 nm, overlapping with the size range of α-amylase self-aggregates. Furthermore, components above 700 nm indicate the formation of PET–α-amylase complexes and imply an interaction between α-amylase and the PET surface. In addition to hydrophobic and van der Waals interactions, PET may enable π–π interactions, dipole–dipole interactions, and hydrogen bonding due to its aromatic rings and polar ester groups, accordingly.^17,21^

The hydrodynamic diameters of the pure PLA particles and the PLA–α-amylase mixture are shown in Figure 1D. The pure PLA particles displayed a broad distribution ranging from approximately 200 to 650 nm, with a peak around 350 nm. This range overlapped notably with that of α-amylase, whose distribution spans 150–700 nm. In the PLA–α-amylase mixture, particle sizes between 150 and 800 nm were observed. Due to substantial overlap among the three distributions, the specific particle–enzyme interaction and subsequent adsorption can be assessed only to a limited extent, suggesting a relatively low affinity. However, minor additional components above 600 nm were observed in the mixture, absent in the individual samples, indicating the formation of some PLA–α-amylase complexes.

### 3.2 Particle-Induced Structural Changes of α-Amylase

To investigate structural and functional changes of adsorbed α-amylase on nanoplastics, secondary structures were analyzed by FTIR spectroscopy (Figure 2), tertiary structures by intrinsic fluorescence spectroscopy (Figure 3), and catalytic function by enzymatic activity assays (Figure 4).

**Figure 2:**
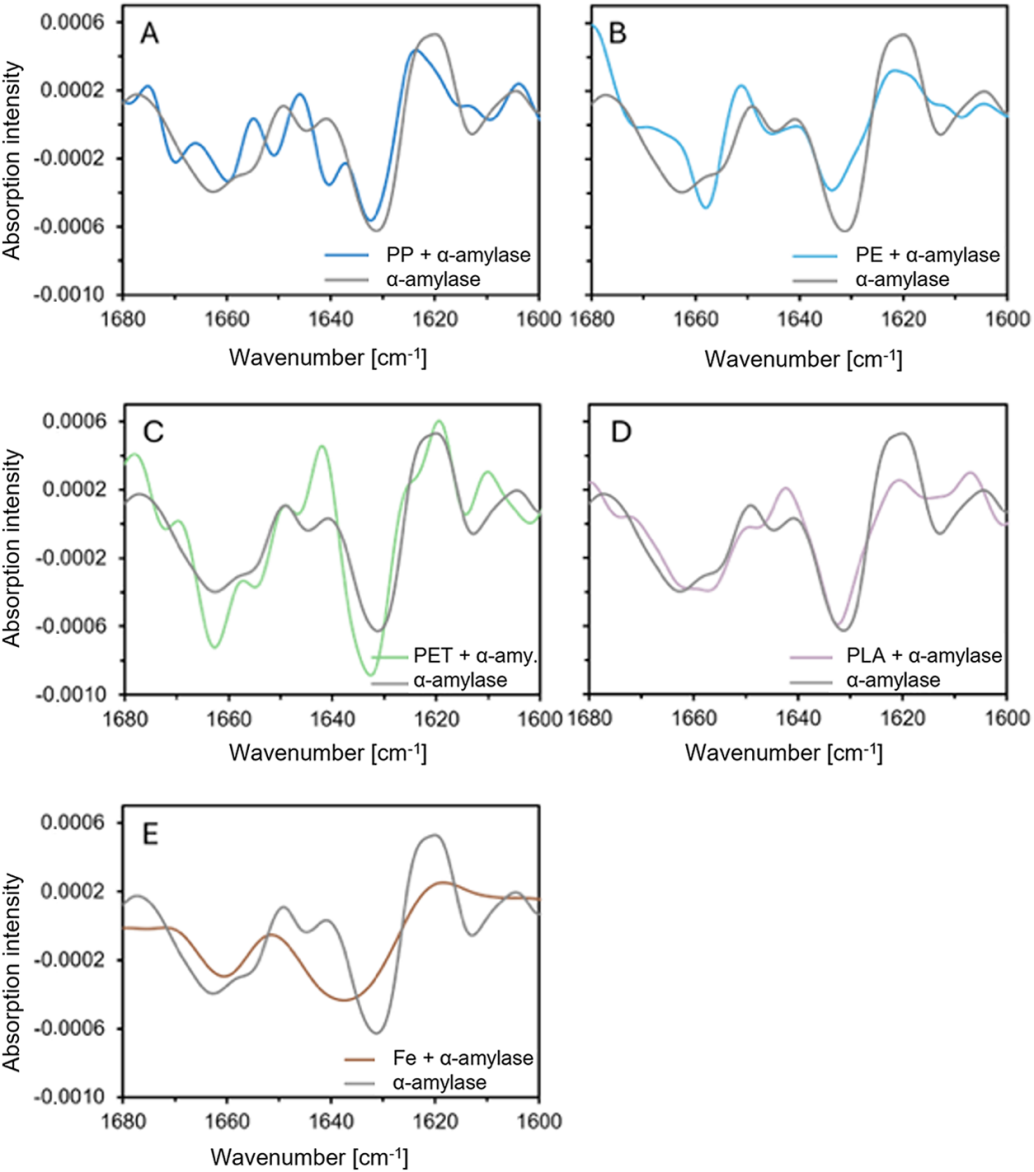
Representation of the second derivative of FTIR spectra of α-amylase in the presence of different nanoplastics and Fe_2_(SO_4_)_3_: (A) PP, (B) PE, (C) PET, (D) PLA, and (E) Fe_2_(SO_4_)_3_ display absolute intensities.

**Figure 3:**
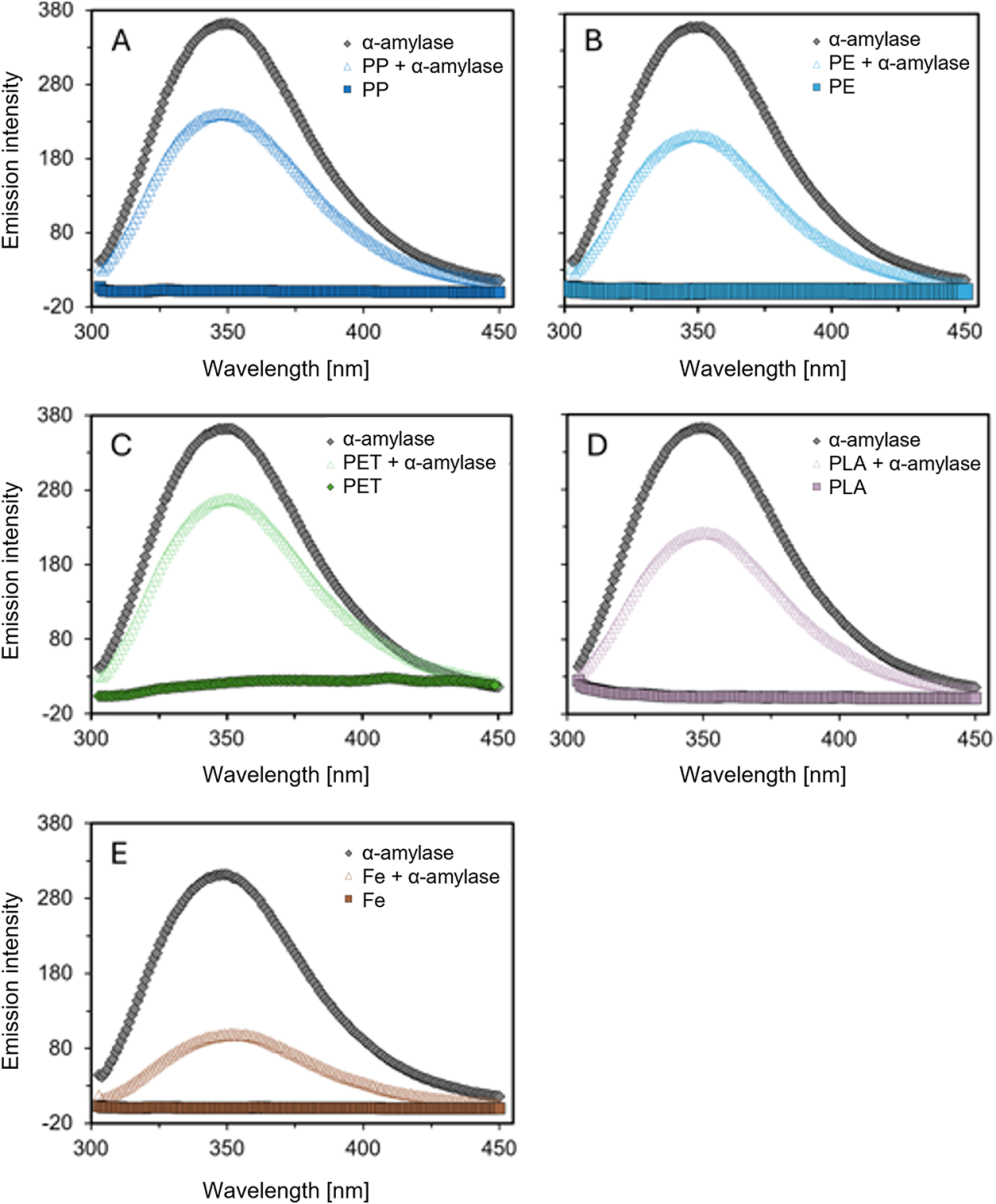
Fluorescence emission spectra of α-amylase in the presence of (A) PP, (B) PE, (C) PET, (D) PLA, and (E) Fe_2_(SO_4_)_3_. The excitation wavelength was 295 nm.

**Figure 4:**
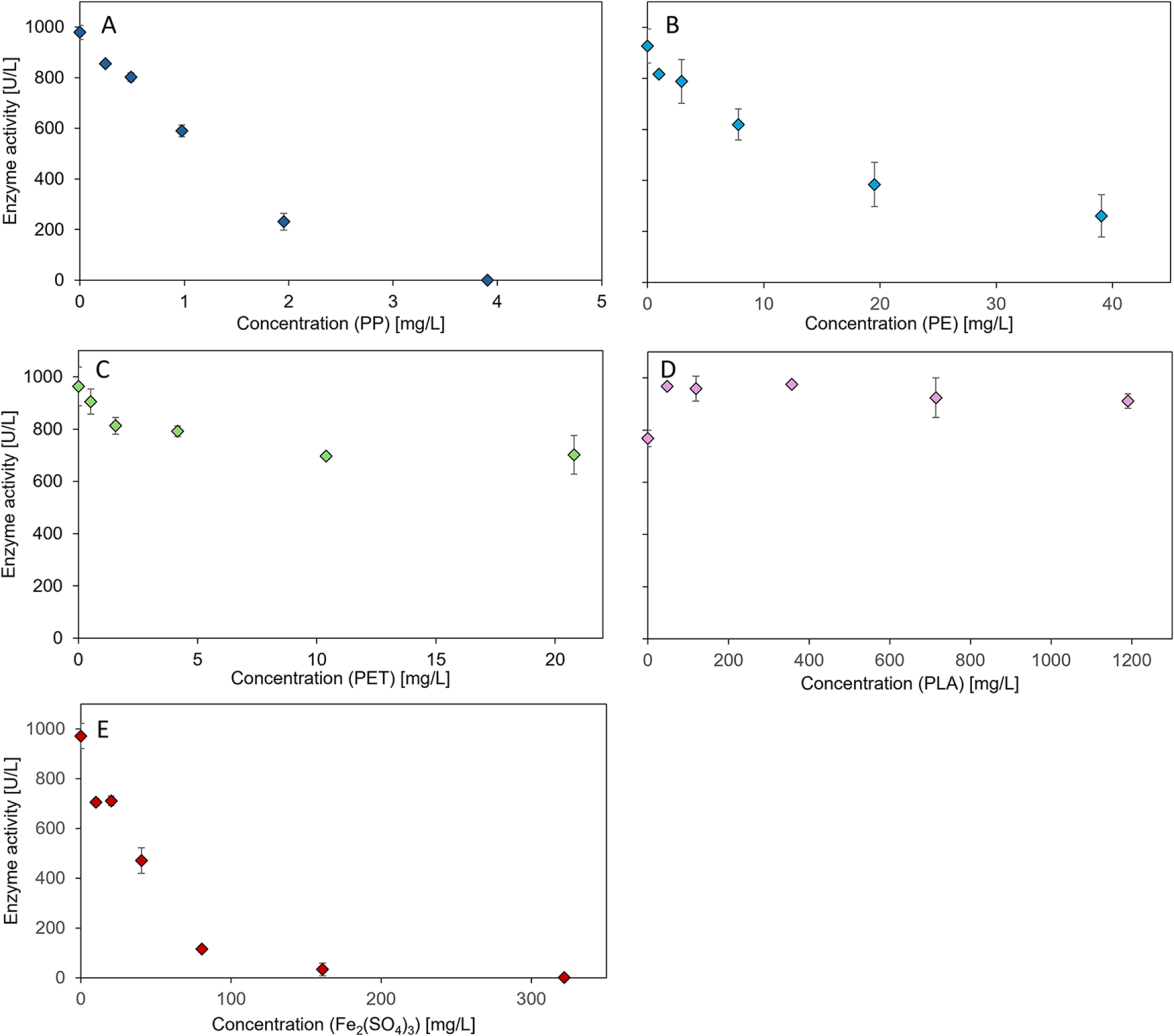
Enzyme activity of α-amylase in the presence of (A) PP, (B) PE, (C) PET, (D) PLA, and (E) Fe_2_(SO_4_)_3_ measured by Phadebas amylase activity test. Enzyme activity, defined as the amount of catalytically formed product in a defined time period, is measured as specific Phadebas substrate turnover, resulting in absorption at 620 nm, measured by UV spectrometer and calculated from a supplied standard curve (Section 2.2.4). Constant amounts of α-amylase were incubated with various nanoplastics concentrations. The variance of concentration ranges is resulting from the differences in particle stock concentrations, shown in Table 1.

In Figure 2, FTIR spectra of the α-amylase structure are dominated by β-structures, with a strong intramolecular β-sheet band at 1631.9 ± 0.6 cm^–1^ and a slightly less intense α-helix band at 1662.6 ± 1.6 cm^–1^ representing the second derivative of FTIR spectra of α-amylase in the presence of different nanoplastics and Fe_2_(SO_4_)_3_. Panels (A) PP, (B) PE, (C) PET, (D) PLA, and (E) Fe_2_(SO_4_)_3_ displayed absolute intensities (Figure 2 and Table S2). In the random coil region, two adjacent bands appeared at 1647.0 ± 2.5 cm^–1^ and 1641.5 ± 0.5 cm^–1^. Such shoulders or double-band features in the amide I region, as described for other proteins like human serum albumin,^24^ likely reflect the coexistence of distinct disordered structures or overlapping contributions from turns and loosely packed β-structures. Overall, the secondary structure composition is consistent with previous studies, which showed that α-amylase contains roughly equal proportions of α-helix, β-structures (including β-sheets and turns), and random coil.^25,26^ Structurally, α-amylase folds into a conserved triosephosphate isomerase (TIM) barrel (also termed α/β-barrel), consisting of eight β-strands surrounded by eight α-helices.^27^ The catalytic site lies within the central TIM-barrel pocket and is essential for both substrate binding and overall enzyme stability.^28^ The active site of α-amylase comprises three aspartate and one glutamate residues that cooperatively hydrolyze the α-(1,4)-glycosidic bond. Each binding subsite interacts with a glucose moiety, typically via aromatic residues (i.e., tryptophan, phenylalanine, tyrosine) that engage in hydrophobic π–π interactions with the sugar rings.^27^ Regarding the tertiary structure, α-amylase showed strong intrinsic fluorescence (Figure 3 and Table S3), consistent with its high aromatic amino acid content (tryptophan, tyrosine, and phenylalanine^28^), with an emission maximum at 362.7 ± 2.4 nm. Finally, the initial enzyme activity was measured approximately, 1000 U/L (Figure 4), confirming robust catalytic function prior to particle interaction.

For the positive control with Fe_2_(SO_4_)_3_, the α-helix component of α-amylase remained stable, indicating that helical structures were largely preserved (Figure 2 and Table S2). In contrast, the random coil double band merged into a single broad band at 1651.1 ± 0.3 cm^–1^ with reduced intensity, suggesting a shift toward more unfolded, flexible conformations. This shift is likely driven by electrostatic and coordination interactions between Fe^3+^ and backbone carbonyls, potentially disrupting intramolecular hydrogen bonding. Similarly, structural effects by divalent cations (Mg^2+^ and Zn^2+^) have been reported by Wei et al. In their study, reduced protein backbone stability of α-amylase was observed by circular dichroism.^29^ Fe^3+^ also strongly reduced intramolecular β-sheet structures, indicative of partial unfolding, which is consistent with denaturation mechanisms previously described for plant-derived α-amylases.^30^ In addition, a significant blue shift of the intramolecular β-sheet was detected, reflecting weaker hydrogen bonds involving backbone carbonyl groups and a loosening of the overall enzyme structure.^31^ Furthermore, a strong decrease in intermolecular β-sheets was observed, highlighting disruption of aggregation-related structures. Regarding the tertiary level, Fe^3+^ caused the largest fluorescence decrease, from 312.2 ± 5.0 to 99.1 ± 2.1 (Figure 3E). In comparison, Mg^2+^ and Zn^2+^ were shown to slightly increase the exposure of Trp-83, located near the active site, but without strong quenching.^29^ No significant shift in the emission maximum was observed, suggesting that quenching results from static complexation or local unfolding rather than complete tertiary refolding. Functionally, Fe^3+^ strongly inhibited α-amylase, leading to complete inactivation at 321.90 mg/mL (Figure 4E). In line with this, nonspecific inhibitors or aggregating particles have been shown to alter hydrogen bonding and π–π interactions near the catalytic site, reducing enzyme efficiency.^29^ Thus, the severe activity loss mirrors extensive secondary and tertiary structure disruptions, likely involving coordination with backbone carbonyls, displacement of catalytically required Ca^2+^, and interference with active site residues. To evaluate the possibility of an inner filter effect, absorbance and fluorescence measurements of the plastic particles alone were performed under the same experimental conditions and concentrations used in this study, including all relevant blank samples. PP and PE exhibited very weak to essentially negligible absorbance at 280 nm, while PLA showed weak to moderate absorbance at 280 nm, clearly lower than PET, which displayed a moderate absorbance due to a broad absorption band extending from approximately 240 to 300 nm (data not shown). Overall, the absorbance of the investigated polymers at 280 nm was low, suggesting that a potential inner filter effect would be minor under the applied conditions.

Upon PP interaction, the α-helix band of α-amylase remained intact compared to the unadsorbed enzyme (Figure 2A and Table S2), indicating that helical structures were largely unaffected. The absence of a wavenumber shift further suggests that no substantial rearrangements of backbone hydrogen bonds occurred in these structures.^31^ The random coil bands showed blue shifts, reflecting local backbone rearrangements and potential overall loosening of the structure, similar to the positive control (Figure 2). By contrast, the intramolecular β-sheet band remained at its initial wavenumber and intensity, although an additional adjacent band at slightly higher wavenumber appeared—potentially attributed loosely packed and flexible β-sheet elements. The intermolecular β-sheet remained unchanged, in contrast to the strong reduction observed for the positive control and PE. At the tertiary structure level, PP reduced fluorescence intensity of α-amylase (Figure 3A), consistent with altered microenvironments of aromatic residues, primarily tryptophan.^32^ Functionally, PP caused a concentration-dependent decrease in activity, leading to full inactivation already at approximately 4 mg/mL (Figure 4A). Enzyme inhibition, caused by polymer particles, was previously shown by Liu et al., who detected mild decreases of enzyme activities of digestive enzymes caused by PVC microplastics particles.^33^ Here, the strong inhibition occurred despite largely preserved secondary and tertiary structures, indicating that adsorption on PP affects the proximity of the catalytic center itself, thereby hindering substrate access to the active site—an inhibition mechanism distinct from Fe^3+^, where potentially unfolding itself drove inhibition. This strong inhibition is consistent with the almost complete adsorption of α-amylase onto PP observed in the hydrodynamic measurements (c.f. Section 1), reflecting high-affinity binding that hinders substrate access to the active site.

For PE, the α-helix band of α-amylase remained unchanged, indicating that helical structures are largely preserved (Figure 2 and Table S2). However, unlike PP, PE substantially increased the first random coil band, reflecting a shift toward more unordered structures. The intramolecular β-sheet band displayed a slight blue shift, similar to the trend observed for the positive control, while its intensity markedly decreased, consistent with partial unfolding and a sheet-to-coil transition. These results agree with descriptions by Azhagesan et al., who reported an α-amylase unfolding upon exposure to polystyrene nanoplastics.^34^ The intermolecular β-sheet content decreased significantly, suggesting reduced aggregation tendency. At the tertiary level, PE induced the strongest fluorescence quenching of all tested particles (Figure 3B), in line with pronounced effects on aromatic residues and local unfolding. Functionally, PE impaired a concentration-dependent loss of activity, though less severe than PP and positive control, with residual activity still detectable at a particle concentration of 39.05 mg/mL (Figure 4). This intermediate inhibition mirrors the moderate but widespread structural alterations and agrees with docking predictions of polystyrene binding near Asp197 and Asp300, disrupting both substrate binding and global conformation^34^ and is moreover consistent with the small fraction of α-amylase bound to PET observed in hydrodynamic measurements, indicating low-affinity interactions.

In contrast to the positive control, PP, and PE, PET enhanced α-helix band intensity (Figure 2 and Table S2), indicating helix formation upon α-amylase adsorption. Similar helix-mediated adsorption mechanisms have been described for serum albumin by Zhang et al. (2023), where helices actively participate in surface adsorption before partially unfolding at later stages.^35^ Random coil structures remained largely unaffected, whereas intramolecular β-sheet intensity significantly increased, consistent with a partial refolding process. This observation parallels earlier findings for β-lactoglobulin adsorbed on PET, where adsorption stabilized certain β-sheet elements.^17^ The intermolecular β-sheet content remained unchanged, similar to PP, indicating aggregation-related structures were preserved. PET caused the weakest fluorescence quenching among all particles (Figure 3), suggesting comparatively mild tertiary structure changes. Moreover, enzyme activity was moderately reduced (Figure 4), consistent with limited α-helix changes, potentially in the TIM-barrel region, and moderate alterations of the tertiary structure. The ability of PET to promote partial refolding while retaining catalytic function contrasts sharply with the positive control, PP, and PE, where unfolding correlated with stronger inhibition.

For PLA, the α-helix band was unaffected, confirming that helical regions are stable upon adsorption (Figure 2D and Table S2). The lower-wavenumber random coil band became more pronounced, reflecting subtle rearrangements in unordered structures. In contrast, intramolecular β-sheets remained stable in both position and intensity, while the intermolecular β-sheet content decreased significantly, suggesting reduced aggregation. Comparable decreases in intermolecular β-sheets have been reported for β-lactoglobulin adsorbed on PLA.^17^ On the tertiary level, PLA caused a pronounced reduction in fluorescence intensity (Figure 3), reflecting quenching of aromatic residues and local rearrangements. Despite these secondary and tertiary alterations, the enzymatic activity was fully preserved (Figure 4), indicating that PLA triggers structural rearrangements away from the active site, allowing catalysis to remain intact—aligning with the low adsorption affinity suggested in Section 3.1. However, this overall trend contrasts with the positive control, PP, and PE, where similar or even less pronounced quenching coincided with strong activity loss, underscoring the spatial specificity of adsorption effects on function.

### 3.3 Cell Biological Investigation of Particle–Enzyme Complexes on Caco-2 Cells

#### 3.3.1 Cell Proliferation and Cell Viability

Based on the discussed adsorption affinities in Section 3.1 and structural findings in Section 3.2, questions arose regarding the possible impact of particle–enzyme complexes on human cells. Therefore, the well-established *in vitro* system Caco-2 was applied to simulate the human intestinal cell layer.^36,37^ Cell growth and viability studies were performed with the pure MNP particles and their complexes with α-amylase. Furthermore, the question was addressed whether the formation of these complexes can impact the uptake of nanoplastics into Caco-2 cells.

To observe the impact on cell growth and cell viability, Caco-2 cells were applied in the xCELLigence system to investigate changes in their cell impedance, which would indicate an impact on cellular parameters.^38^ The resulting cell index is proportional to changes in electrical resistance. It increases with the number of cells, cell adhesion, and spreading, serving as an indirect indicator of cell proliferation or cell viability. The study involved applying pure α-amylase, pure nanoplastics (PP, PE, PET, PET-Alexa, and PLA), and mixtures of α-amylase and nanoplastics to the cells. The incubation lasted over 72 h, and the cell index was continuously recorded throughout. Zinc chloride served as a toxic positive control. After a resting time of around 24 h, Caco-2 cells start to proliferate exponentially until they reach confluency, which can be observed by measuring their cell index. Figure 5A shows the increasing cell indices of proliferating Caco-2 cells. α-Amylase was applied in a 1:10 dilution, which was toxic to proliferating Caco-2 cells, indicated by a reduction of cell indices. Cytotoxic impact of α-amylase was previously observed in other mammalian cell types.^39,40^ In contrast, cells incubated with α-amylase 1:100 showed normal growth. Therefore, nanoplastics were incubated alone and in complex with α-amylase 1:100. All conditions indicated normal growth curves for nanoplastics as well as nanoplastics–amylase complexes, using α-amylase in a 1:100 dilution. The slight differences are all within the range of normal deviation. Zn^+^ was used as a positive control, leading to a rapid reduction of cell indices in accordance with previous experiments.^17,41,42^ The cell index measurements were also performed on differentiated Caco-2 cells as a measure for cytotoxicity (Figure 5B). For differentiated Caco-2 cells, α-amylase was nontoxic also in a 1:10 dilution, which allowed the use of higher concentrated nanoplastics–amylase complexes with α-amylase in 1:10 dilution. Taken together, none of the nanoplastics species led to cytotoxicity and there were no differences between pure particles and particle–protein complexes.

**Figure 5:**
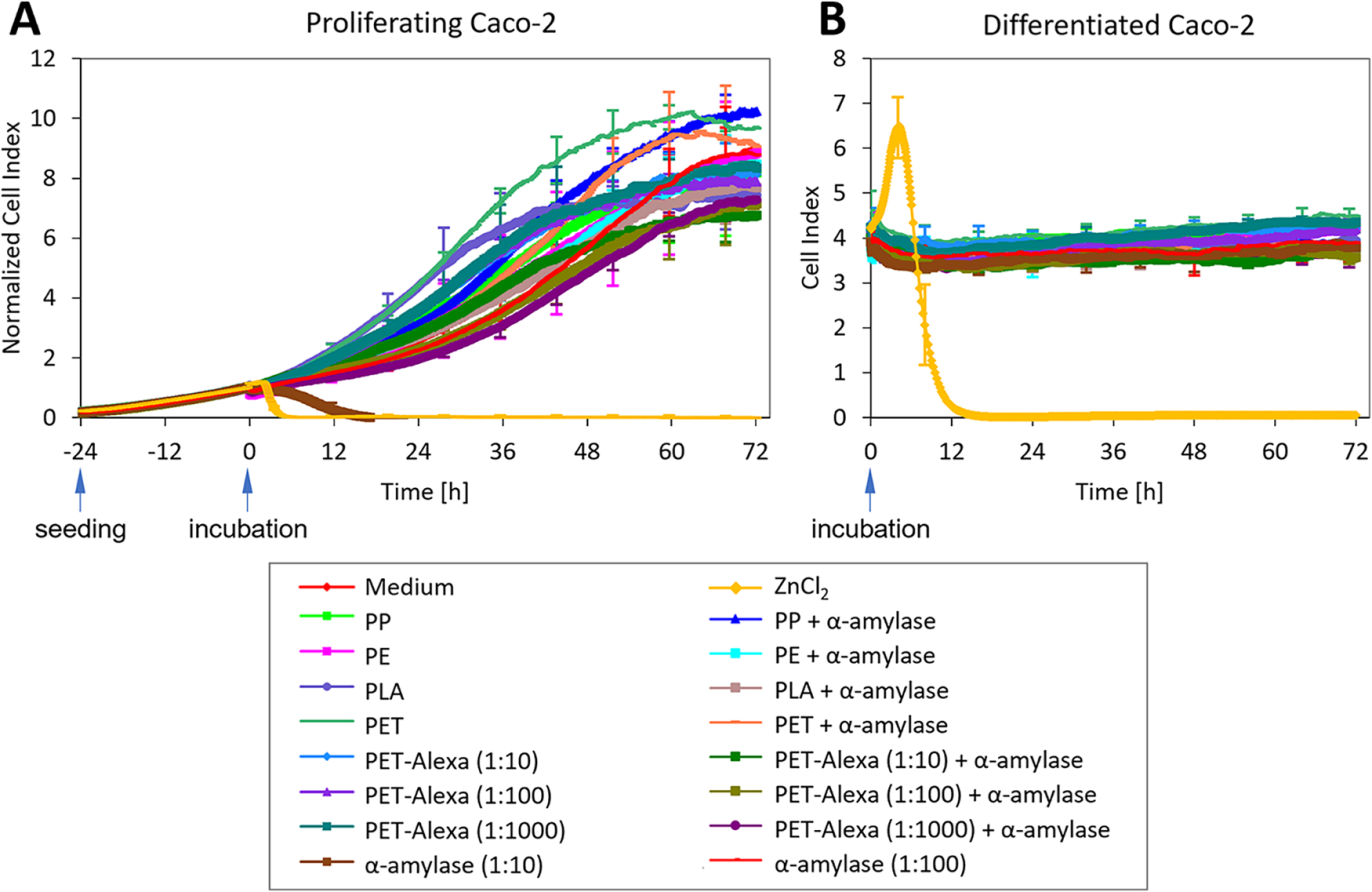
Cell impedance measurements via the xCELLigence method. (A) Cell proliferation of undifferentiated Caco-2 cells over a time period of 72 h after incubation with particles and complexes. Cell indices were normalized on the time point 30′ prior to incubation. (B) Cell viability of differentiated Caco-2 cells over a time period of 72 h after incubation with particles and complexes. Mean values were determined from 3 technical replicates ± std. dev.

#### 3.3.2 Uptake of Particle–Enzyme Complexes into Caco-2 Cells

Besides toxicology testing, a major question is whether enzyme binding can influence the uptake of nanoplastics into the cells. Different methods need to be considered, depending on whether the particles contain a fluorescence label or not. For nonlabeled particles, cellular uptake of MNP can be observed by optical microscopy in brightfield mode. However, this is only possible for certain MNP depending on the size and refractive properties of the particles and the optical resolution of the microscope, which is limited by the Abbe-limit in the range of 300–500 nm size.^43^ Larger particle agglomerations can be visible as well as structural changes of cell morphologies after particle uptake, often materializing in granular inclusions. These morphological changes can also be detected by flow cytometry, leading to increased side-scatter values.^17,42,44^ In the present study, the applied nonfluorescent nanoplastics caused no changes in cell morphologies (Figure S1). Images of cells incubated with PP and PLA showed the presence of particle agglomerates, indicated by dark spots, while the presence of α-amylase did not lead to differences. With this method, particle identity as well as internalization cannot be confirmed. Furthermore, increased side-scatter signals were visible neither for proliferating nor for differentiated Caco-2 cells (Figure S2). The slight increase for proliferating Caco-2 cells with α-amylase (1:10) can be reasoned by the onset of apoptosis. Consequently, uptake of nonlabeled particles could not be proven using side-scatter analysis. Also, the particle–α-amylase complexes did not show differences in the outcome, compared to pure nanoplastics particles.

As label-free techniques did not lead to a meaningful outcome about MNP uptake, we applied a fluorescence label, using Alexa-633 dye, as exemplified here with the PET particles. The Alexa-633 staining has been applied before as a high possibility to visualize the particles in an *in vitro* system-compatible way.^45^ Many dyes, such as Nile Red, tend to leach in the context with biological media.^46^ Others, mainly green fluorescent dyes, can interfere with cellular autofluorescence. So, this red fluorescent, stable-linked fluorophore is particularly well-suited for the upcoming *in vitro* experiments. After labeling, cleanup, and purification, as described in the methods section, the functionality was confirmed by measuring absorption and fluorescence spectra of the labeled particles (Figure S3). Fluorescence intensity scans showed that the wavelengths of 610/650 nm (ex/em) provided the highest signal-to-noise and were therefore used for all further experiments.

The use of fluorescence to detect particle uptake can be carried out by a variety of methods. Here, a setup of microscopic as well as spectroscopic methods was applied (Figure 6). Using Cell Discoverer, the presence of fluorescent particles and agglomerates in the cell layer can be observed. Red fluorescence was visible across the cell layer in the PET-Alexa containing samples, which was not present in the nonlabeled ones (Figure 6A). However, due to resolution limitations, these spots are more likely agglomeration than single particles. In addition, simple fluorescence microscopy cannot give a three-dimensional resolution, which means that it was not possible to discriminate between internalized and externally attached particles. A fluorescence scan over the cell layer using a Tecan plate reader (Figure 6B) showed a concentration-dependent increase in fluorescence values for PET-Alexa particles. Also, the mean values increased concentration-dependently using PET-Alexa complexes with α-amylase in a comparable quantity. As control, nonlabeled particles and α-amylase alone did not cause increased signals. Particle uptake can also be observed using flow cytometry, where MNP uptake leads to increased fluorescence signals in the respective channel. Figure 6C shows representative flow cytometer plots where a notable right-shift of the population in the matching fluorescence channel (FL4, Ex./Em. 640/675 nm) is visible. Here, the mean values also increased concentration-dependently using PET-Alexa particles and complexes with α-amylase (Figure 6D). In contrast to previous studies with larger microparticles, a determination of the particle number was not possible using flow cytometry.^44^ The right-shift of the whole population only indicated a general interaction of the cell population with the fluorescently labeled particles, where it is not possible to discriminate between particle internalization and external attachment, though. Nonlabeled particles showed a low autofluorescence, and α-amylase alone did not cause increased signals. However, in both assays and for both cell types, proliferating (Figure S4) and differentiated Caco-2 cells (Figure 6D), here the presence of α-amylase caused slightly lower fluorescence signals, compared to nanoplastics applied alone, but this effect was not supported by statistical significance. The slight decrease could be caused by a coverage of the particles by the enzyme adsorption layer (Section 3.1), leading to fewer interactions with the cell surface, which was observed before for other nanomaterials.^12^

**Figure 6:**
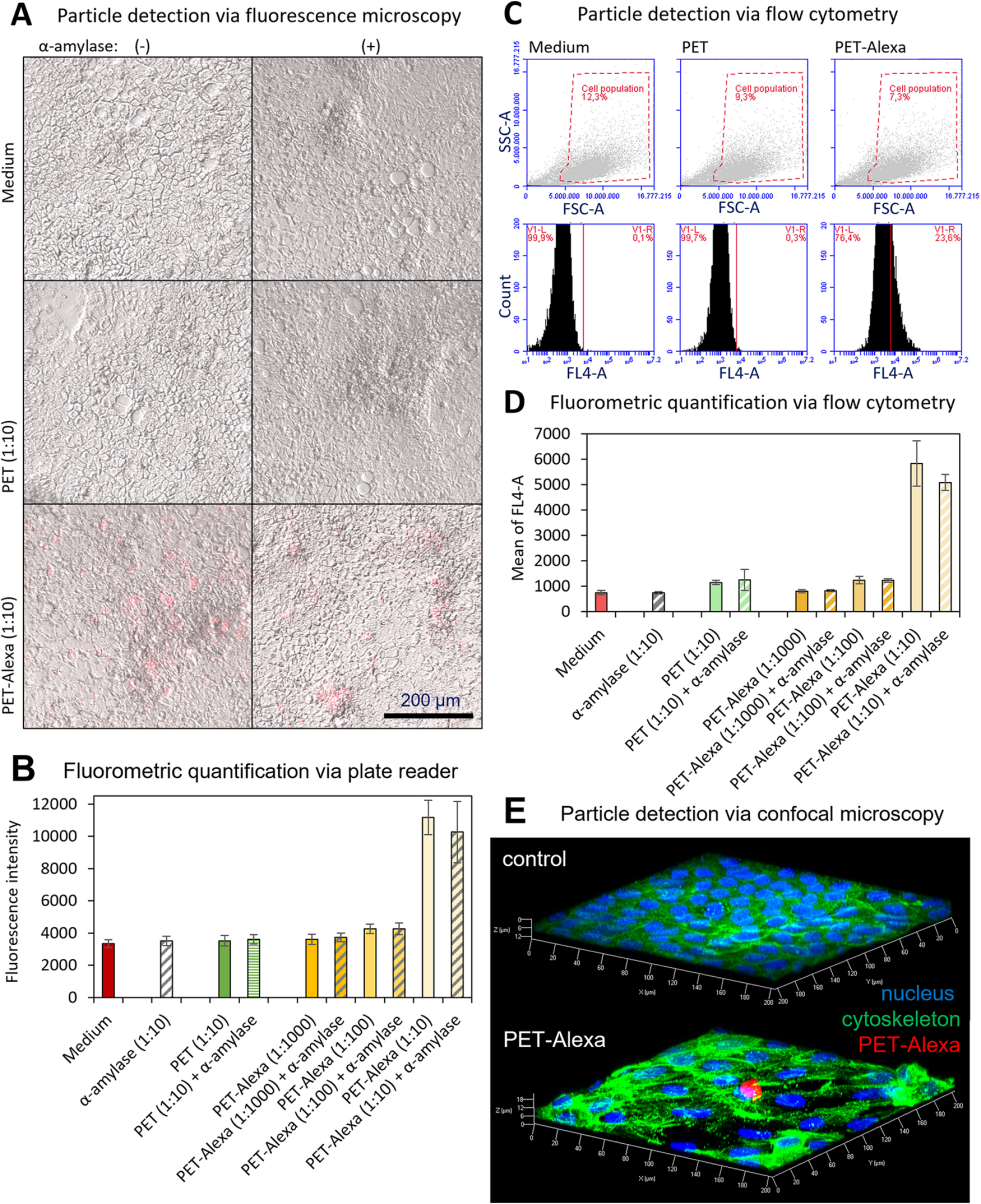
Fluorometric measurements of particle uptake into differentiated Caco-2 cells after 24 h incubation with PET-Alexa with and without α-amylase complexes. (A) Optical microscopy images taken on viable cells after thorough washing with PBS. Acquisition in brightfield mode merged with fluorescence detection (Ex./Em.: 631/647 nm). (B) Fluorometric quantification of the same samples using plate reader in scanning mode (Ex./Em.: 610/650 nm). Mean values were determined from at least 3 independent experiments, each using at least 3 technical replicates ± Std. Dev. (C) Exemplary FSC/SSC scatter plots and FL4 fluorescence intensity plots using flow cytometry (Ex./Em.: 640/675 nm). (D) Fluorometric quantification of FL4 mean signals in the flow cytometer (Ex./Em.: 640/675 nm). Mean values were determined from at least 3 independent experiments, each using at least 2 technical replicates ± std. dev. (E) Confocal microscopy of differentiated Caco-2 cells after 24 h incubation with PET-Alexa and medium control after fixation and fluorometric staining as described. Images were taken as z-stacks using the fluorescence channels DAPI (Ex./Em.: 405/435 nm), Actin Green (Ex./Em.: 488/518 nm), and PET-Alexa (Ex./Em.: 639/669 nm).

Finally, cells were scanned for nanoplastics uptake using a confocal fluorescence microscope. Cell nuclei and cytoskeleton (actin) were stained in blue and green to enable the possibility of particle localization within three-dimensional cell visualizations. In the present study, the detection of single particles in cell compartments was not possible when using this method. With the use of this method, it became clear that only the detection of agglomerates attached to the outer cell layer was possible, as shown in an exemplary 3D image in Figure 6E. PET-Alexa was only detected in one sample on the cell surface, not inside the cells. In all other samples, no red fluorescence was detected, suggesting that the particles adsorb only to the cell surface and are predominantly washed away during multiple washing steps (which were necessary for nuclear and actin filament staining). Taken together, the setup of experiments with fluorescent labeled particles did not indicate particle internalization, independent of the question whether the nanoplastics were applied alone or in complex with α-amylase.

## 4 Conclusion

This study addressed the relevance of molecular interactions between enzymes and nanoplastics by examining how the model enzyme α-amylase interacts with different model polymer types (PP, PE, PET, and PLA), providing mechanistic insight into how these initial molecular interactions may affect the enzyme structure, function, and cellular responses. Adsorption of α-amylase occurred for all nanoplastics, with stronger binding observed for the more hydrophobic polymers PP and PE, and weaker interactions for PET and PLA. Structural analyses showed that interactions with PP and PE induced more pronounced structural rearrangements, whereas PET tended to stabilize specific structural elements, and PLA caused only minor alterations. These particle-dependent structural responses only partially explained changes in enzyme activity: the observed inhibition was largely determined by adsorption affinity rather than the magnitude of structural changes, with PP and PE causing the strongest inhibition, PET a moderate reduction, and PLA maintaining activity. This trend highlights the relevance of local interactions near the active site and the role of binding strength in modulating enzyme function. Cell experiments using Caco-2 cells indicated that both particles and their enzyme complexes were nontoxic; although interactions with the cell surface were observed, no measurable internalization occurred for any particle type. To further support mechanistic understanding and extend beyond previously published studies, PET nanoplastics were labeled with Alexa fluorescent dye, improving localization in cells and enabling more precise tracking of particle–enzyme complexes.

Taken together, these findings suggest that direct nanoplastics–enzyme interactions constitute an initial molecular event that can influence enzyme structure and function, potentially further propagating to cellular-level effects in complex biological systems. The results provide a mechanistic framework for understanding how nanoplastics may impact such biological systems and offer a basis for future hazard and risk assessments. Further experiments should explore how these molecular interactions affect a broader range of enzymes, including those present within cells, investigate the influence of particle size and surface chemistry, and assess their potential long-term cellular effects.

## Supplementary Material



## Notes

Preparation of reagents and reaction solutions (Methods); overview of the intensity-weighted hydrodynamic diameters (Table S1); summary of the intensity maxima and minima and the corresponding wavenumbers of FTIR spectra (Table S2); summary of the wavelengths of intensity maxima of fluorescence emission (Table S3); representative microscopic overview images (Figure S1); cell granularity analysis (Figure S2); absorption and fluorescence spectra of Alexa-633-stained and unstained PET particles (Figure S3); and fluorescence intensity plots (PET-Alexa-633) for proliferating Caco-2 cells in flow cytometry (Figure S4) (PDF)
