# Supplementary material for: Unraveling Nanoplastics–Enzyme Interactions: Physicochemical, Structural, Functional, and Cell Biological Characterization of α‑Amylase–Nanoplastics Complexes
Source: Langmuir. 2026 Jul 7;42(28):20153–65. doi: 10.1021/acs.langmuir.6c00976 (PMC13394412; doi:10.1021/acs.langmuir.6c00976)
Supplement: Supplementary file 1 [file la6c00976_si_001.pdf]

# Unraveling Nanoplastics-Enzyme Interactions: Physicochemical, Structural, Functional, and Cell Biological Characterization of α-Amylase-Nanoplastics Complexes

## - Supporting Information -

Holger Sieg[a*], Franziska Ott[b], Linda Böhmert[a], Stephan Drusch[c], Andreas F. Thünemann[d], Sascha Rohn[b], Helena Kieserling[b],

[a]*Department of Food and Feed Safety in the Food Chain, Unit Novel Foods, GMOs, Food Additives, Flavourings and Feed Additives, German Federal Institute for Risk Assessment (BfR), Max-Dohrn-Str. 8-10, 10589 Berlin, Germany*
[b]*Department of Food Chemistry and Analysis, Institute of Food Technology and Food Chemistry, Technische Universität Berlin, Kaiserin-Augusta-Allee 14, 10553 Berlin, Germany*
[c]*Department of Food Technology and Material Science, Institute of Food Technology and Food Chemistry, Technische Universität Berlin, Straße des 17. Juni 135, 10623 Berlin, Germany*
[d]*Bundesanstalt für Materialforschung und -prüfung (BAM), Unter den Eichen 87, 12205 Berlin, Germany*

Holger Sieg*: holger.sieg@bfr.bund.de, Franziska Ott: ott.3@campus.tu-berlin.de, Linda Böhmert: linda.boehmert@bfr.bund.de, Stephan Drusch: stephan.drusch@tu-berlin.de, Andreas Thünemann: andreas.thuenemann@bam.de, Sascha Rohn: rohn@tu-berlin.de, Helena Kieserling: helena.schestkowa@tu-berlin.de

*Corresponding author: holger.sieg@bfr.bund.de

**Supporting Information: Materials and Methods**

**Preparation of α-amylase solutions**

In this study, a variety of spectroscopic and analytical assays were performed, each of which, however, required specific concentrations and other pre-requisites. The composition of each of them is described as follows:

**Solution for dynamic light scattering (DLS).** For DLS measurements, a 2% by weight α-amylase solution was prepared. Alpha-amylase (2.00 g) was dissolved in 100 mL of $CaCl_2$ solution (1.89 mg/mL). The mixture was then sonicated in an ultrasonic bath for 10 min at a temperature not exceeding 22 °C, followed by stirring with a magnetic stirrer for at least 2 h. This α-amylase solution will be called α-amylase solution 1.

**Solution for fluorescence and fourier-transform infrared spectroscopy (FTIR) spectroscopy.** For fluorescence and FTIR spectroscopy, 1% w/w α-amylase solutions were prepared. In α-amylase solution 2, 0.2 mg of α-amylase was dissolved in 20.0 mL of $CaCl_2$ solution, resulting in a concentration of 1.89 mg/mL. In α-amylase solution 3, 0.1 mg of α-amylase was dissolved in 10.0 mL of $CaCl_2$ solution, yielding a final concentration of 1.89 mg/mL. The solutions were then pre-vaporized and sonicated in an ultrasonic bath for 15 minutes.

**Solution for the α-amylase activity test.** An α-amylase solution (5.71 mg/mL) was prepared by dissolving 0.114 g of α-amylase in 10.0 mL of $CaCl_2$ solution (1.89 mg/mL). The solution was vortexed for 30 s and then filtered through a polyvinylidene fluoride (PVDF) filter (200 nm). For further use, aliquots of 1–1.5 mL were transferred into 2 mL reaction vessels and stored at −11 °C in a freezer. On the day of the experiment, an aliquot was thawed by hand, pre-textured, and then stored in a refrigerator at 8 °C until use. For activity testing, the α-amylase solution was diluted 1:20 by mixing 500 μL of the α-amylase solution (5.71 mg/mL) with 9.50 mL of $CaCl_2$ solution (1.89 mg/mL). This 1:20 dilution (0.57 mg/mL) is referred to below as α-amylase solution 4.

**Solution for cell biology experiments.** Saturated α-amylase solutions were prepared for cell biology experiments. To make the solution, two spatula tips of α-amylase were added to a sterile 5 mL reaction vessel and mixed with 4 mL of sterile ultrapure water. The mixture was vortexed for 30 s and stored overnight at 4 °C. The next day, the solution was vortexed again for 30 s and left to stand for 5 min until a complete sediment formed. The amount needed was then carefully taken from the supernatant for the experiments. The α-amylase solution used in the cell biology experiments is referred to below as α-amylase solution 5.

**Calcium chloride solution.** A $CaCl_2$ solution (1.89 mg/mL) was prepared by dissolving 0.189 g of $CaCl_2$ in ultrapure water (100 mL, pH 5.84). The pH was then adjusted to 7 with 0.01 M NaOH using a pH meter.

**Iron(III) sulfate solution.** $Fe_2(SO_4)_3$ (0.338 g) was dissolved in ultrapure water (20.0 mL). The initial pH (5.84) was then adjusted to 7 with 0.01 M NaOH, as measured with a pH meter. A 1:10 dilution of the $Fe_2(SO_4)_3$ solution was prepared by mixing 1.0 mL of the $Fe_2(SO_4)_3$ solution with 1.0 mL of ultrapure water (pH = 7). The 1:10 dilution (1.69 mg/mL) is referred to below as the Fe3+ solution.

## Supporting Information: Results

Table S1: Overview of the intensity-weighted hydrodynamic diameter of pure α-amylase, free nanoplastics, and mixtures of α-amylase and nanoplastics. Shown are the mean values of triplicate measurements and the standard deviation of the individual results. Letters at mean values indicate a statistically significant difference (p < 0,05, ANOVA, and Tukey's Test).

| | Mean | SD |
|---|---|---|
| α-amylase | $404^{bc}$ | 29 |
| PP | $218^{eh}$ | 12 |
| PP+ α-amylase 1:1 | $571^{a}$ | 89 |
| PP+α-amylase 1:10 | $469^{ab}$ | 30 |
| PE | $188^{gh}$ | 14 |
| PE +α-amylase 1:1 | $202^{fh}$ | 22 |
| PE + α-amylase 1:10 | $255^{dh}$ | 41 |
| PET | $150^{h}$ | 45 |
| PET +α-amylase 1:1 | $389^{bd}$ | 60 |
| PET + α-amylase 1:10 | $360^{bde}$ | 10 |
| PLA | $395^{bd}$ | 21 |
| PLA+α-amylase 1:1 | $336^{bdef}$ | 24 |
| PLA + α-amylase 1:10 | $300^{cdefg}$ | 33 |

Table S2: Summary of the intensity maxima and minima and the corresponding wavenumbers of the FTIR spectra of α-amylase, the α-amylase-nanoplastics mixtures and α-amylase-Fe mixture in the amide I band, assigned to the different secondary structure elements. Calculated mean values (MW) and standard deviations (STABW) are shown. Different letters show a statistically significant difference ($p < 0.05$, ANOVA followed by Tukey's test or Kruskal-Wallis test followed by Dunn's test).

| | Wavenumber [$cm^{-1}$] | | Absolute intensity | |
|---|---|---|---|---|
| **α-helix** | | | | |
| α-amylase | $1662.6^{ab}$ | ± 1.6 | $-0.00041^{a}$ | ± 0.00002 |
| PP + α-amylase | $1656.4^{a}$ | ± 4.1 | $-0.00028^{a}$ | ± 0.00006 |
| PE + α-amylase | $1658.0^{ab}$ | ± 0.1 | $-0.00042^{a}$ | ± 0.00005 |
| PET + α-amylase | $1663.2^{b}$ | ± 1.9 | $-0.00063^{b}$ | ± 0.00008 |
| PLA + α-amylase | $1656.7^{ab}$ | ± 0.5 | $-0.00039^{a}$ | ± 0.00002 |
| Fe + α-amylase | $1660.0^{ab}$ | ± 0.5 | $-0.00031^{a}$ | ± 0.00003 |
| **random-coil** | | | | |
| α-amylase | $1647.0^{c}$ | ± 2.5 | $0.00009^{b}$ | ± 0.00001 |
| | $1641.5^{bc}$ | ± 0.5 | $0.00008^{a}$ | ± 0.00003 |
| PP + α-amylase | $1654.4^{a}$ | ± 0.4 | $0.00004^{b}$ | ± 0.00002 |
| | $1645.8^{a}$ | ± 0.2 | $0.00017^{a}$ | ± 0.00006 |
| PE + α-amylase | $1650.2^{bc}$ | ± 0.8 | $0.00019^{a}$ | ± 0.00005 |
| | $1641.3^{c}$ | ± 0.8 | $0.00006^{a}$ | ± 0.00007 |
| PET + α-amylase | $1649.3^{bc}$ | ± 0.6 | $0.00011^{ab}$ | ± 0.00000 |
| | $1642.0^{bc}$ | ± 0.0 | $0.00030^{a}$ | ± 0.00013 |
| PLA + α-amylase | - | - | - | - |
| | $1643.2^{b}$ | ± 0.7 | $0.00015^{a}$ | ± 0.00005 |
| Fe + α-amylase | $1651.1^{ab}$ | ± 0.3 | $-0.00005^{c}$ | ± 0.00003 |
| | - | - | - | - |

| | | | | |
|---|---|---|---|---|
| **intramolecular β-sheet** | | | | |
| α-amylase | 1631.9$^{a}$ | ± 0.6 | -0.00063$^{c}$ | ± 0.00002 |
| PP + α-amylase | 1632.5$^{ab}$ | ± 1.3 | - 0.00060$^{bc}$ | ± 0.00002 |
| PE + α-amylase | 1633.9$^{b}$ | ± 0.4 | - 0.00049$^{ab}$ | ± 0.00008 |
| PET + α-amylase | 1632.1$^{a}$ | ± 0.4 | -0.00083$^{d}$ | ± 0.00004 |
| PLA + α-amylase | 1632.4$^{a}$ | ± 0.4 | - 0.00058$^{bc}$ | ± 0.00001 |
| Fe + α-amylase | 1637.1$^{c}$ | ± 0.5 | -0.00042$^{a}$ | ± 0.00001 |
| **intermolecular β-sheet** | | | | |
| α-amylase | 1621.1$^{ab}$ | ± 1.0 | 0.00050$^{ab}$ | ± 0.00005 |
| PP + α-amylase | 1623.5$^{a}$ | ± 0.3 | 0.00043$^{bc}$ | ± 0.00004 |
| PE + α-amylase | 1621.3$^{ab}$ | ± 0.6 | 0.00036$^{cd}$ | ± 0.00003 |
| PET + α-amylase | 1620.7$^{ab}$ | ± 2.1 | 0.00056$^{a}$ | ± 0.00003 |
| PLA + α-amylase | 1620.7$^{ab}$ | ± 0.1 | 0.00029$^{d}$ | ± 0.00004 |
| Fe + α-amylase | 1618.0$^{b}$ | ± 0.4 | 0.00026$^{d}$ | ± 0.00002 |

Table S3: Summary of the wavelengths of the intensity maxima of the fluorescence emission. The calculated mean values (MEAN) and standard deviations (SD) are shown. Different letters show a statistically significant difference ($p < 0.05$, Kruskal-Wallis test followed by Dunn's test).

| | Wavelenghts [nm] of the intensity maxima | | | | | | |
|---|---|---|---|---|---|---|---|
| | α-amylase 2 | PP + α-amylase 2 | PE + α-amylase 2 | PET + α-amylase 2 | PLA + α-amylase 2 | α-amylase 3 | Fe + α-amylase 3 |
| | 350 | 350 | 347 | 350 | 348 | 346 | 351 |
| | 350 | 349 | 349 | 350 | 350 | 349 | 354 |
| | 352 | 347 | 349 | 352 | 351 | 350 | 351 |
| MEAN | 350.7$^{a}$ | 348.7$^{a}$ | 348.3$^{a}$ | 350.7$^{a}$ | 349.7$^{a}$ | 348.3$^{a}$ | 352.0$^{a}$ |
| SD | 0.9 | 1.2 | 0.9 | 0.9 | 1.2 | 1.7 | 1.4 |

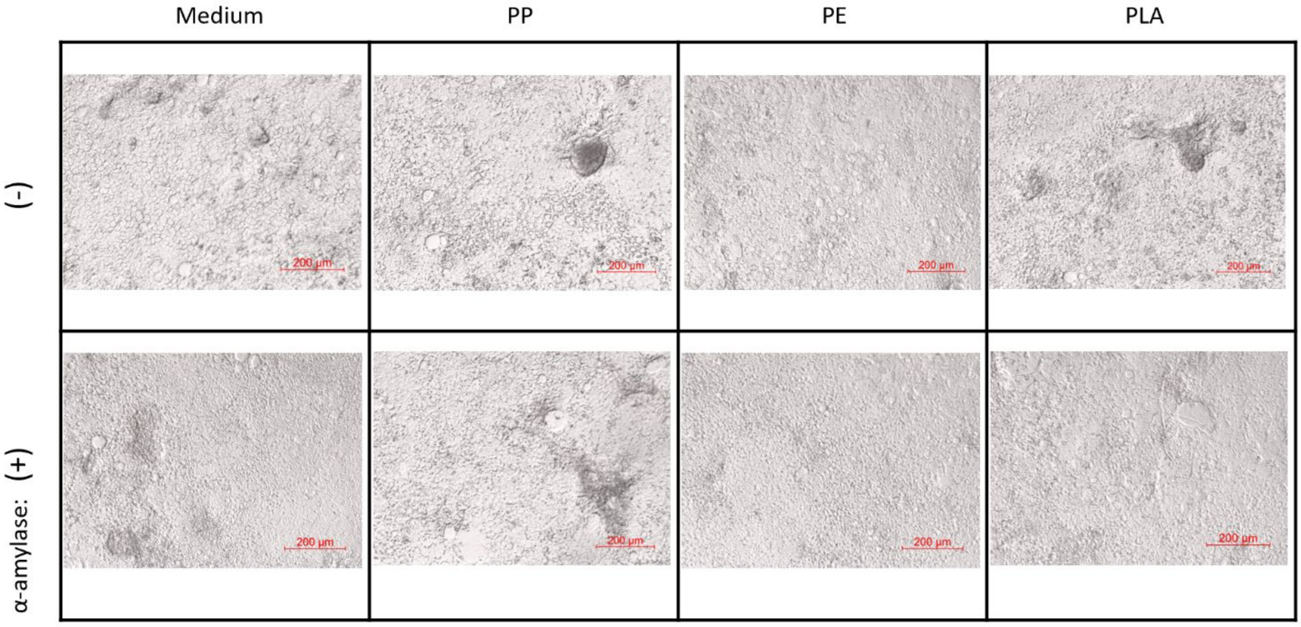


Figure S1: Representative overview images (magnification 50 x), of non-fluorescent particles, taken by optical microscopy by cell discoverer, brightfield mode.

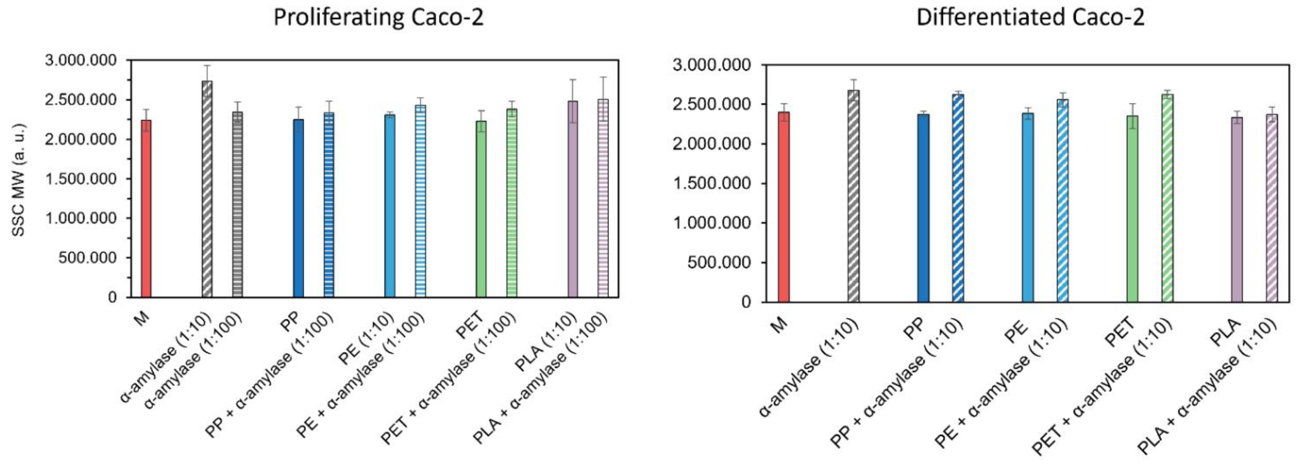


Figure S2: Cell granularity analysis, done by flow cytometry. Side scatter (SSC) intensity mean values indicated by std. dev. of at least 2 replicates in 3 independent experiments.

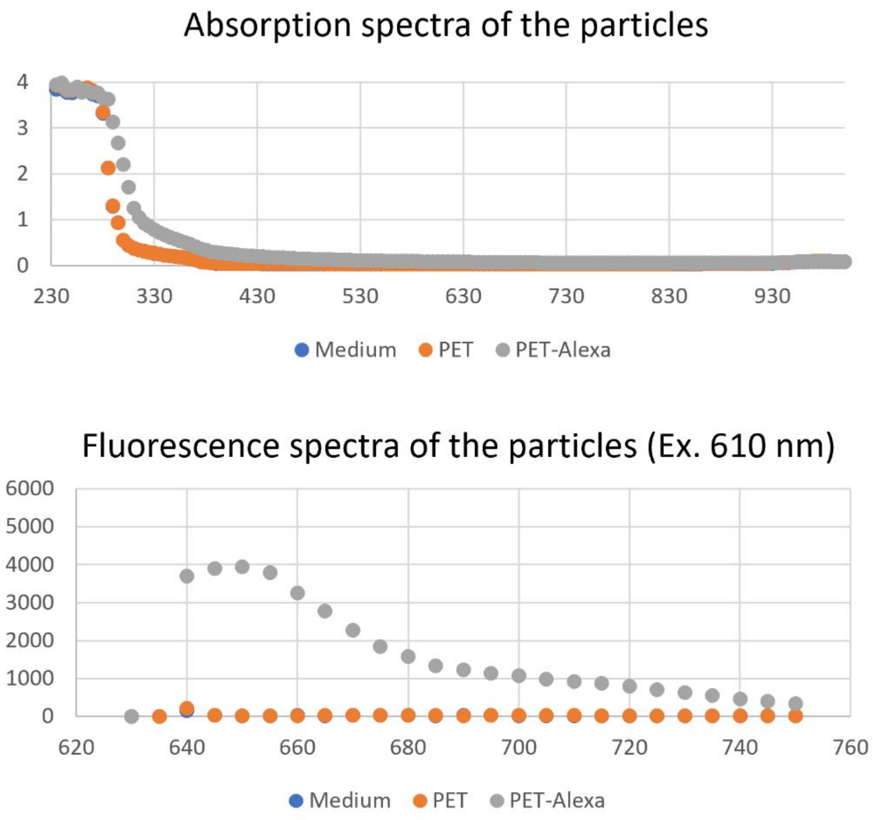


Figure S3: Absorption and fluorescence spectra of Alexa-633-stained and unstained PET particles and controls. A: Absorption spectra in an observation range between 230 and 1000 nm. B: Fluorescence intensity spectra, measured with an excitation wavelength of 610 nm in an observation range (emission) between 620 and 760 nm.

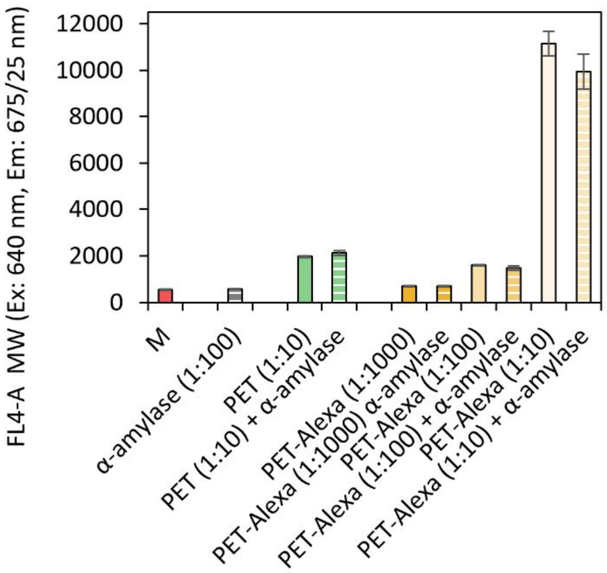

Figure S4: Fluorescence intensity (Ex. 640 nm, em. 675/25 nm) measured by flow cytometry. Results given for proliferating Caco-2 cells incubated with nanoplastics, nanoplastics-enzyme-complexes and controls. Results given as mean values indicated by std. dev. of at least 2 replicates in 3 independent experiments.
